# Hematuria was a high risk for renal progression and ESRD in immunoglobulin a nephropathy: a systematic review and meta-analysis

**DOI:** 10.1080/0886022X.2021.1879852

**Published:** 2021-03-09

**Authors:** Peng He, Hanmin Wang, Chen Huang, Lijie He

**Affiliations:** Department of Nephrology, State Key Laboratory of Cancer Biology & Institute of Digestive Diseases, Xijing Hospital, the Fourth Military Medical University, Xi’an, China

**Keywords:** Hematuria, IgAN, renal outcomes, systematic review, meta-analysis

## Abstract

**Background:** The relationship between hematuria, a typical presentation of immunoglobulin A nephropathy (IgAN), and long-term adverse prognosis of these patients is still controversial. This meta-analysis aims to clarify the effect of hematuria on renal outcomes in IgAN.

**Methods:** Observational cohort studies reporting associations between various forms of hematuria and renal outcomes among IgAN patients were identified from the PubMed and Embase databases. The pooled adjusted risk ratios (RRs) were computed with random effects models.

**Results:** Thirteen studies encompassing 5660 patients with IgAN were included. Patients with initial hematuria did not have a significantly increased risk of developing end-stage renal disease (ESRD) compared with those without hematuria (RR, 1.32; 95% CI, 0.87–2.00; *p* = .19). However, initial microscopic hematuria was associated with an 87% increase in the risk of ESRD (RR, 1.87; 95% CI, 1.40–2.50; *p* < .001), while macroscopic hematuria was associated with a 32% decrease in the risk of ESRD (RR, 0.68; 95% CI, 0.58–0.79; *p* < .001). Additionally, persistent hematuria might be an independent risk factor for ESRD or a 50% decline in eGFR.

**Conclusions:** Among IgAN patients, hematuria, including initial microscopic hematuria and even persistent hematuria, was possibly associated with renal progression and ESRD. However, independent of other classical predictors, initial macroscopic hematuria might be a protective factor for IgAN.

## Introduction

Immunoglobulin A nephropathy (IgAN) is the most common primary glomerular disease worldwide, especially in East Asian China, and is a major cause of end-stage renal disease (ESRD) in a substantial proportion of patients within 10 to 20 years from its apparent onset [1–3]. It is characterized by recurrent episodes of asymptomatic hematuria with or without proteinuria, hypertension or decreased glomerular filtration rate (GFR) at baseline [2,4–9]. Among these symptoms, hematuria is the most typical presentation of IgAN. Approximately 70% to 100% of patients have asymptomatic microscopic hematuria or macroscopic hematuria, which often occurs after upper respiratory tract or intestinal infection [10].

The long-term outcomes of IgAN patients have conclusively been shown to be impacted by decreased renal function at presentation, hypertension and proteinuria. However, evidence regarding the kidney-related prognosis of IgAN patients who present with normal renal function, isolated microscopic hematuria and minimal or no proteinuria is lacking [1,5,6,11–13]. Hematuria of IgAN not only affects these anxious IgAN patients but also puzzles nephrologists during treatment. Actually, nephrologists devote more attention to the monitoring and therapeutic targeting of another key manifestation of glomerular injury, i.e., proteinuria. It is important to establish whether hematuria of IgAN is a progressive factor and to seek clinical or histologic findings that could predict a worse long-term outcome. Some studies showed that there were no negative effects of hematuria on the outcome of IgAN patients with mild proteinuria or even severe hematuria, so immunosuppressive treatment was not needed. Gutierrez E’s study showed that the long-term prognosis for IgAN patients who present with minor urinary abnormalities and normal renal function is excellent [5]. Another study showed that, in IgAN patients with mild proteinuria (less than 0.5 g/day), the hematuria was naturally decreased without any intensive therapy and severe hematuria was not related with the progression to increasing proteinuria and ESRD [14]. However, data from a small sample in China and Japan suggested that IgAN comorbid with hematuria and minimal proteinuria is usually a progressive disease [5,7,15,16]. More data are needed to identify the renal outcomes and prognosis of hematuria of IgAN.

The aim of our meta-analysis was to systematically and quantitatively review original studies published from January 1, 1990, to May 14, 2020, that examined the impact of hematuria on renal outcomes in patients with IgAN.

## Methods

The present meta-analysis was conducted in accordance with the Preferred Reporting Items for Systematic Review and Meta-Analyses (PRISMA, Appendix 1) and the Meta-Analysis of Observational Studies in Epidemiology (MOOSE) guidelines [17,18].

### Literature search

The PubMed and Embase databases were systematically and independently searched by 2 researchers (P.H. and H.M.W.). Original articles published in English from January 1, 1990, to May 14, 2020, were inspected for eligibility. The following terms were used: IgA Glomerulonephritis, IgA Nephropathy, Immunoglobulin A Nephropathy, IgA Type Nephritis, Berger Disease, IgA Nephropathy 1, hematuria, and hematuria (Appendix 2). Any discrepancy was resolved by discussion with a third researcher (L.J.H.). Additional articles were identified from the reference lists of relevant papers and obtained through manual search.

### Study selection

Two levels of screening were performed. The first level was performed by screening titles and abstracts. Articles with information about the prognosis of IgAN patients were included. The second level was performed by screening the full texts of articles to identify studies focused on the associations between various forms of hematuria (i.e., hematuria, microscopic hematuria, macroscopic/gross hematuria, and persistent hematuria) and renal outcomes. Mild hematuria, 1–29 RBCs/HPF or 1+/2+ (dipstick), was considered a form of microscopic hematuria. Persistent hematuria was defined as time-average (TA) hematuria > 5 RBCs/high-power field (HPF). The inclusion criteria were as follows: (a) cohort studies with biopsy-proven IgAN patients; (b) studies with an estimate of the association between hematuria and renal outcomes (i.e., relative risk [RR], hazard ratio [HR], odds ratio [OR]) and 95% confidence intervals (CIs), or relevant data to calculate them; (c) studies with end points that included end-stage kidney disease (ESRD), a 50% decline in estimated glomerular filtration rate (eGFR), or doubling of serum creatinine (SCr) concentration. The exclusion criteria were as follows: (a) elderly or childhood studies and (b) other kinds of articles, including cross-sectional studies, reviews, case reports, letters, editorials, comments, supplements, and conference abstracts. For multiple papers using the same cohort or database, the sample with the most comprehensive and recent data was included.

### Data extraction and quality assessment

Two researchers (P.H. and H.M.W.) independently extracted relevant information from the eligible articles using a standardized data collection form. The extracted data included the first author, publication year, country, study design, demographics (i.e., patient number, initial age [median or mean], and proportion of male gender), 24-h urine protein excretion and estimated glomerular filtration rate at baseline (median or mean), follow-up duration, number of patients with ESRD, hematuric classification, time point of detection, renal outcome, and effect size. The quality of eligible articles was appraised with the Newcastle Ottawa Scale for cohort studies, and scores ranged from 0 to 9. We considered articles with scores < 5 as having a high risk of bias [19]. We attempted to extract data to evaluate the associations between the magnitude of baseline hematuria and ESRD and between hematuria and renal progression among IgAN patients. However, since these data were rarely reported in the studies and the definitions of hematuria varied, we only included a simple description in the subsequent analysis.

### Statistical analysis

Our meta-analysis was conducted using the DerSimonian-Laird random effects model. The effect sizes from the final model that adjusted for the maximum covariates were used from the eligible studies. For studies that reported RRs, the RRs and 95% CIs were extracted directly using participants with outcomes of interest without hematuria as the reference group. HRs were assumed to be numerically the same as the RRs. For studies that did not report HRs, the estimated HRs and 95% CIs were computed by the available Kaplan–Meier curves using the Engauge Digitizer software, version 4.10 and the method of Tierney et al. [20]. If the effect sizes could not be obtained, the crude RRs were calculated by the chi-square test.

Pooled estimates were calculated on the logarithm of the RR from the individual studies. The results were then transformed back to the RR scale. The between-study heterogeneity was examined with Cochran’s Q test. Significant heterogeneity was defined as a value of *p* < .10. The I^2^-statistic was used to quantify the heterogeneity. Jackknife sensitivity analyses were performed by omitting 1 study at a time and repeating the meta-analysis [21]. A two-tailed *p* < .05 was considered statistically significant. Statistical analyses were performed in Stata software, version 15.0 (StataCorp).

## Results

### Study selection

**Figure 1.**
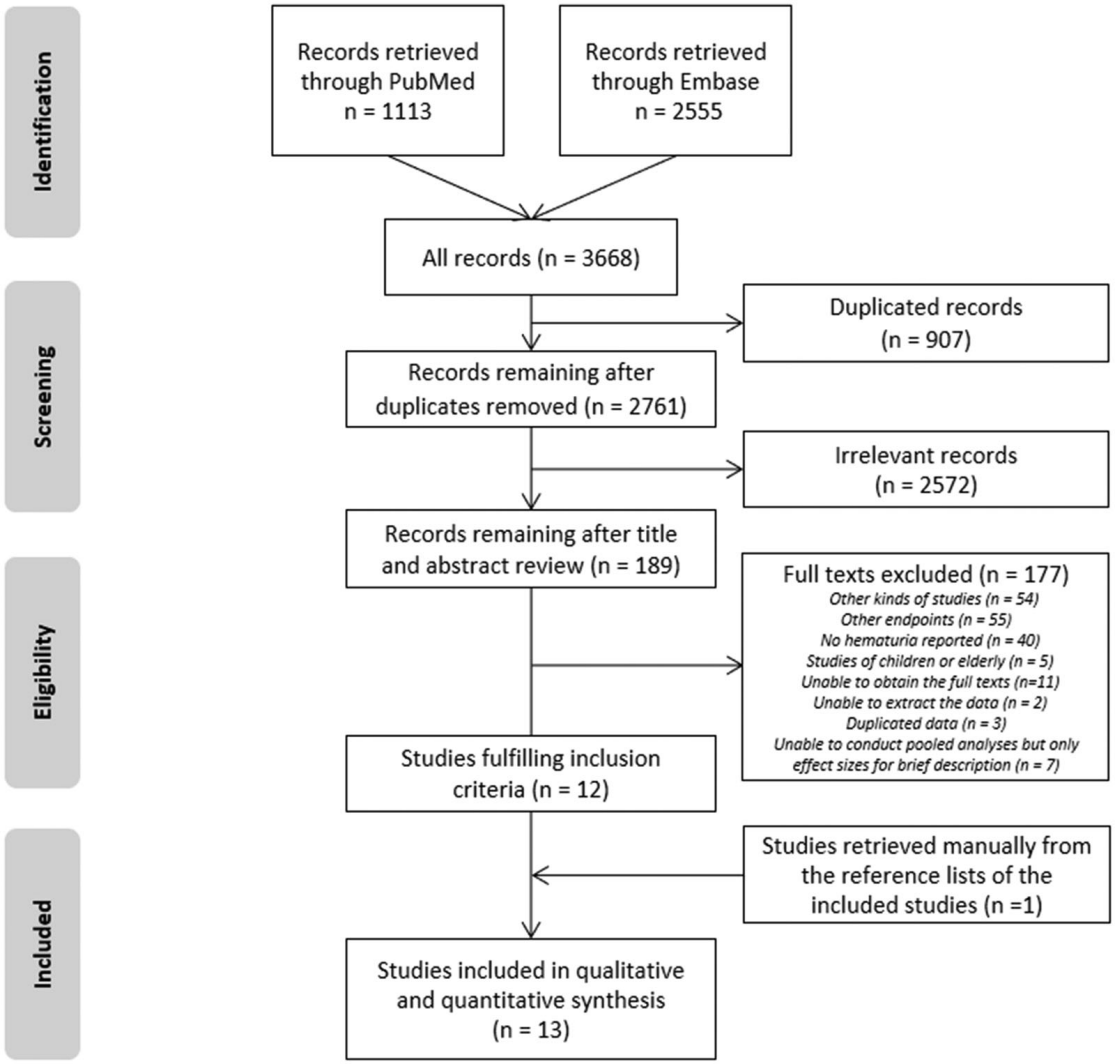
Flow chart of study selection from literature search.

After removing 907 duplicates, the remaining 2761 records were processed, and 12 studies were eligible (Figure 1). Most records (*n* = 2572) were irrelevant, and 177 records were excluded after the full-text assessment. One additional study was retrieved manually from the reference lists of the included studies. Ultimately, a total of 13 articles were included in the meta-analysis [9,14,22–32].

### Study characteristics and quality assessment

The study and participant characteristics are summarized in Table 1. Of the included studies encompassing 5660 patients, 4 studies were from Asia, and 9 were from Europe and America. The median follow-up times were between 3.6 and 10.3 years. The median baseline proteinuria was between 0.3 and 2.9 g/d. There were 2 prospective studies, and the other studies were retrospective. The association between initial hematuria (binary variable) and ESRD was investigated in 7 studies [9,22,23,27,29,30,32]. Among them, 4 studies [9,27,29,32], focused on micoscopic hematuria. The predictive value of macroscopic hematuria for ESRD was evaluated in 7 studies [9,14,24–26,28,31].

**Table 1.** Primary characteristics of included studies.

Reference	Country	Design	Patients, no.	**Age**^a^**, yrs**	Male, %	Protainuria^a^, g/d	**EGFR**^a^**, ml/min per 1.73 m^2^**	**Follow-up**^a^**, yrs**	ESRD, no.	Classification	Time point	Outcomes
De Menezes et al. [22]	Brazil	RCS	111	32	30.6	2.0	53	5.3	31	Hematuria	Biopsy	SCr doubling/ESRD
Bobart et al. [23]	USA	RCS	72	44.8	67.2	2.5	54.6	3.7	9	Hematuria	Biopsy	ESRD
Heybeli et al. [24]	Turkey	RCS	100	42.1	64	1.7	67	6.3	14	Macroscopic hematuria	Biopsy	SCr doubling/50% decline in eGFR/ESRD
Tanaka et al. [14]	Japan	RCS	88	33.9	38.6	0.3	89.4	NR	NR	Macroscopic hematuria	Biopsy	ESRD
Le et al. [25]	China	RCS	1155	34	49.7	0.9	89.7	7.9	155	Macroscopic hematuria	Before biopsy	ESRD
Bjørneklett et al. [27]	Norway	RCS	633	39	74	NR	67	10.3	146	Microscopic hematuria	Biopsy	ESRD
Lee et al. [26]	Korea	RCS	1364	33	50	1.3	67.6	8	277	Macroscopic hematuria	Biopsy	ESRD
Goto et al. [9]	Japan	PCS	2283	NR	48.7	NR	NR	7	207	Macroscopic and microscopic hematuria	Biopsy	ESRD
Espinosa et al. [28]	Spain	RCS	59	31.6	74.6	2.4	68.4	NR	11	Macroscopic hematuria	Biopsy	ESRD
Manno et al. [29]	Italy	RCS	437	31	67.7	0.7	NR	9	72	Microscopic hematuria	Onset	ESRD
Daniel et al. [30]	France	RCS	194	37.8	75.8	NR	NR	3.6	32	Hematuria	Biopsy	ESRD
Haas [31]	USA	RCS	109	38.4	73.4	2.9	NR	NR	NR	Macroscopic hematuria	Before biopsy	ESRD
Frimat et al. [32]	France	PCS	210	36.2	82.3	1.6	NR	5.6	33	Microscopic hematuria	Onset	ESRD

^a^Median or mean.

eGFR: estimated glomerular filtration rate; ESRD: end-stage renal disease; NR: not reported; PCS: prospective cohort study; RCS: retrospective cohort study; SCr: serum creatine level.

The eligible studies were of moderate quality, as indicated by their scores of 5-8 points on the Newcastle Ottawa Scale (Appendix 3). Seven studies [14,22,25–28,30] lacked a statement on the adequacy of follow-up, and the follow-up rates in 6 studies [9,23,24,29,31,32] were lower than 90%. The median follow-up of 2 studies [23,30] was less than 5 years, while another 3 studies [14,28,31] lacked relevant data. Additionally, the outcome assessment of 1 cohort [31] came from the results of questionnaires.

### Initial hematuria and ESRD

Seven studies assessed the association between initial hematuria (binary variable, vs. negative) and ESRD (*n* = 3940). The corresponding pooled RR was 1.32 (95% CI, 0.87–2.00; *p* = .190; I^2^ = 72%). The results of the jackknife sensitivity analyses are presented in Appendix 4. The pooled RRs were not significantly changed with each sequential study exclusion. This suggested that the pooled result was robust and not skewed by any particular study.

**Figure 2.**
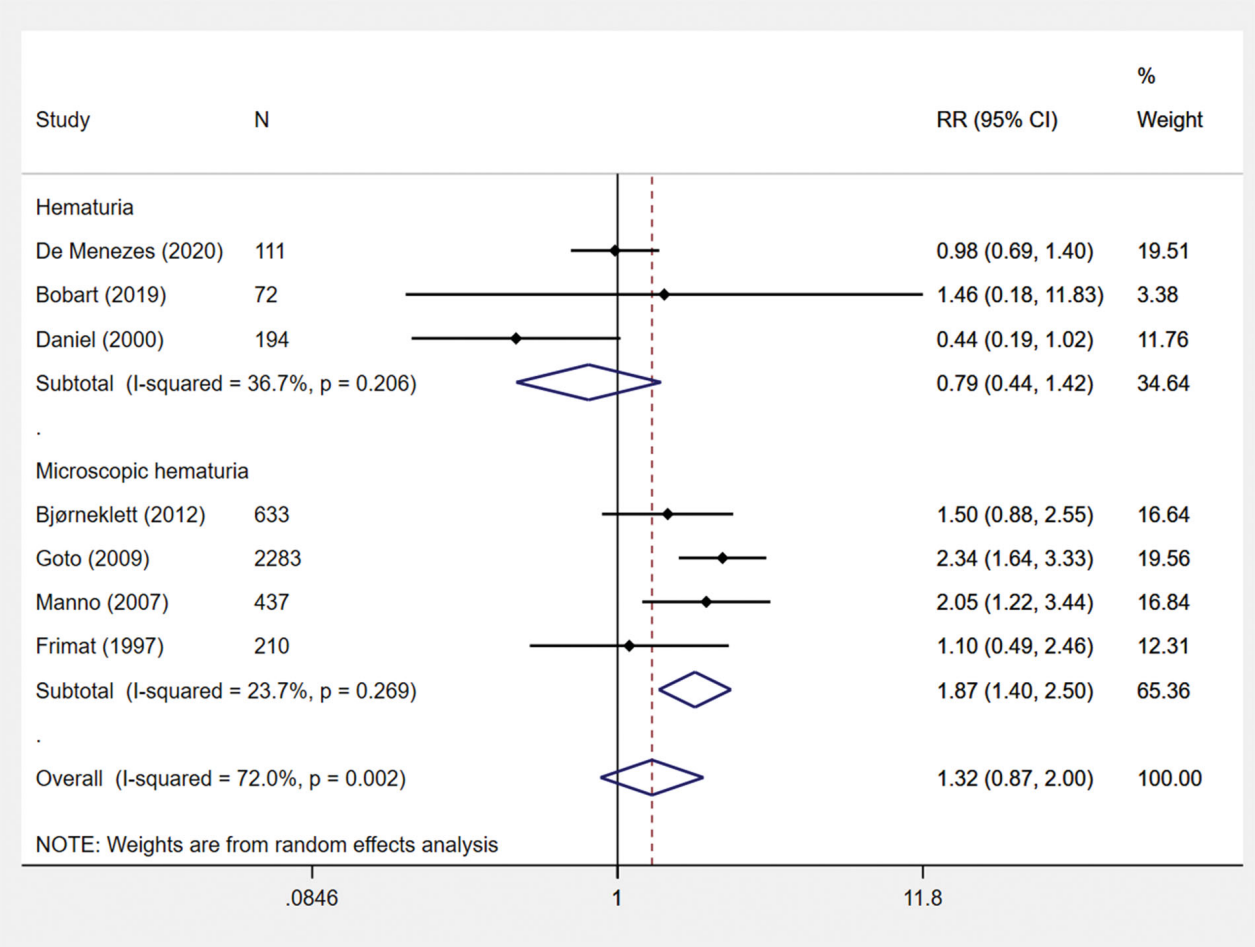
The meta-analysis on the association between initial hematuria and ESRD. CI: confidence interval; ESRD: end-stage renal disease; RR: relative risk.

Four studies assessed the association between microscopic hematuria and ESRD (*n* = 3563). The pooled RR was 1.87 (95% CI, 1.40–2.50; *p* < .001; I^2^ = 23.7%) (Figure 2). Moreover, the pooled RRs for hematuria at renal biopsy (*n* = 3293) and disease onset (*n* = 647) were 1.21 (95% CI, 0.69–2.12; *p* = .503; I^2^ = 97.1%) and 1.63 (95% CI, 0.90–2.93; *p* = .107; I^2^ = 38.5%), respectively. Other subgroup analyses, e.g., European and American studies, studies with proteinuria > 1 g/d, or eGFR < 60 mL/min/1.73m^2^ were also conducted. The results were summarized in Table 2.

**Table 2.** Subgroup analyses with regard to the association between various hematuria and end-stage renal disease.

Classification	Study no.	Patient no.	RR (95% CI)	*p* Value	I^2
Initial hematuria	7	3940	1.32 (0.87, 2.00)	.190	72%
*hematuria*	3	377	0.79 (0.44, 1.42)	.434	36.7%
*Microscopic hematuria*	4	3563	1.87 (1.40, 2.50)	<.001	23.7%
*At biopsy*	5	3293	1.21 (0.69, 2.12)	.503	79.1%
*Disease onset*	2	647	1.63 (0.90, 2.93)	.107	38.5%
*Europe and America*	6	1657	1.16 (0.78, 1.74)	.468	56.5%
*Proteinuria > 1 g/d*	3	393	1.01 (0.73, 1.39)	.962	0%
*EGFR < 60 ml/min/1.73m^2^*	2	183	0.99 (0.70, 1.41)	.961	0%
Initial macroscopic hematuria	7	5158	0.68 (0.58, 0.79)	<.001	12.2%
*At biopsy*	5	3894	0.68 (0.57, 0.82)	<.001	23.2%
*Before biopsy*	2	1264	0.60 (0.41, 0.87)	.008	0%
*Aisa*	4	4890	0.59 (0.46, 0.76)	<.001	0%
*Europe and USA*	3	268	0.70 (0.56, 0.89)	.003	32.2%
*Proteinuria > 1 g/d*	5	3915	0.64 (0.52, 0.80)	<.001	39.5%
*Proteinuria < 1 g/d*	2	1243	0.67 (0.44, 1.00)	.052	0%
*EGFR > 60 ml/min/1.73m^2^*	5	2766	0.73 (0.64, 0.83)	<.001	0%

CI: confidence interval; eGFR: estimated glomerular filtration rate; RR: risk ratio.

Another 3 studies focused on the association between the extent of hematuria and ESRD were identified. In the retrospective cohort of Bobart et al. [23], the degree of hematuria at biopsy was reported as 0, <3, 3–10, 11–20, 21–30, 31–40, 41–50, 51–100 or >100 RBCs/HPF. In the Cox proportional hazard model, the corresponding HR was 0.85 (95% CI, 0.64–1.14; *p* = .28). Of 2 studies from the same center in Japan [14,33], the magnitude of hematuria at biopsy (per 20 RBCs/HPF increase) was evaluated in IgAN patients with proteinuria (> 1 g/day) and mild proteinuria (< 0.5 g/day). The HRs were 0.75 (95% CI, 0.55–1.00; *p* = .053) and 1.16 (95% CI, 0.89–1.46; *p* = .247), respectively.

### Initial macroscopic hematuria and ESRD

**Figure 3.**
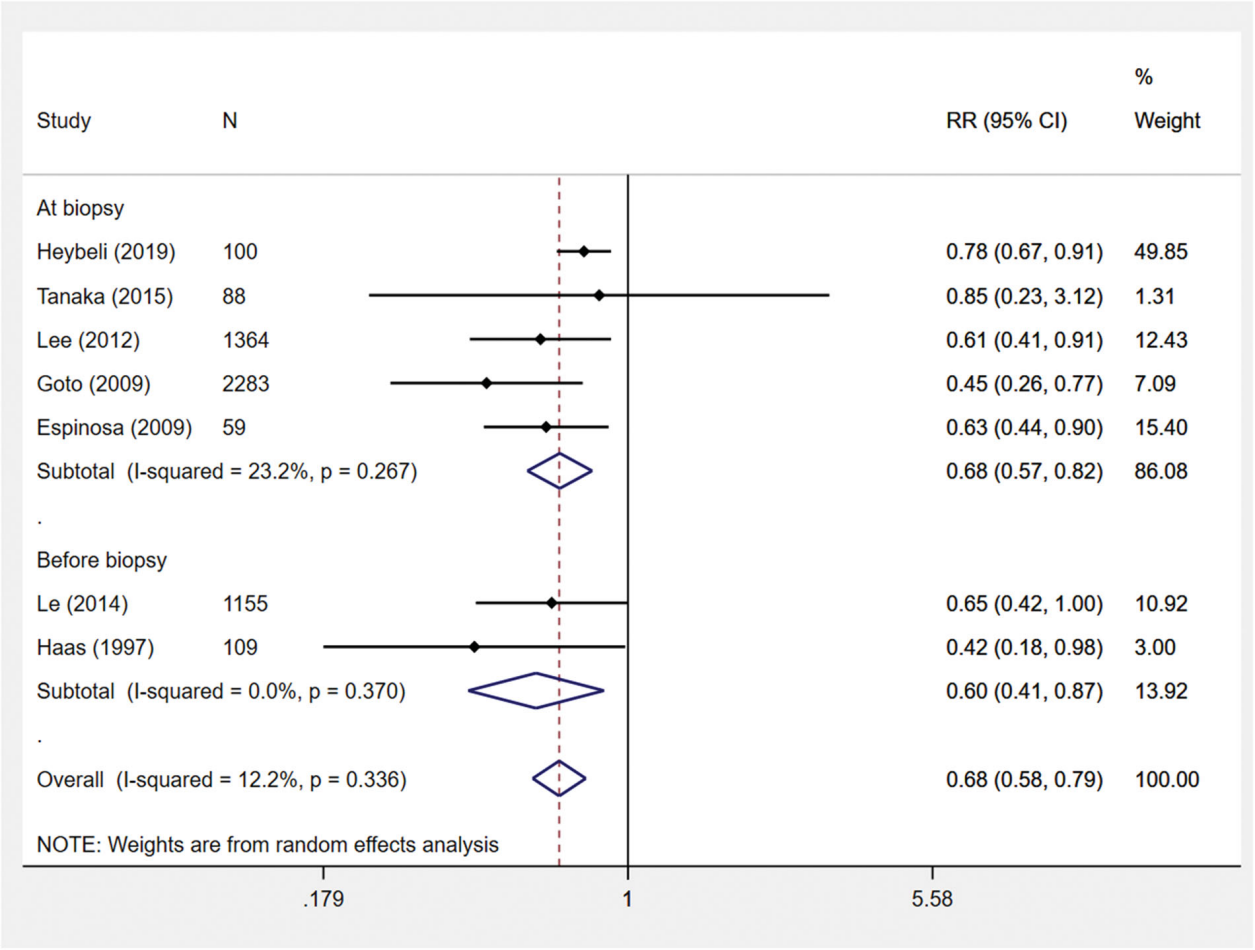
The meta-analysis on the association between initial macroscopic hematuria and ESRD. CI: confidence interval; ESRD: end-stage renal disease; RR: relative risk.

The predictive value of macroscopic hematuria for ESRD was evaluated in 7 studies (*n* = 5158). The meta-analysis suggested that macroscopic hematuria was associated with a decreased risk for ESRD among IgAN patients (RR, 0.68; 95% CI, 0.58–0.79; *p* < .001; I^2^ = 12.2%) (Figure 3). In sensitivity analyses (Appendix 4), the point estimates of the pooled RRs ranged from 0.60 to 0.73 and the corresponding 95% CIs remaining < 1 in all analyses.

Subgroup analyses showed that the pooled RRs for Asian (*n* = 4890) and European/American studies (*n* = 268) were 0.59 (95% CI, 0.46–0.76; *p* < .001; I^2^ = 0%) and 0.70 (95% CI, 0.56–0.89; *p* = .003; I^2^ = 32.2%), respectively. The median proteinuria of 2 studies was less than 1 g/d (*n* = 1243). The pooled RR was 0.67 (95% CI, 0.44–1.00; *p* = .052; I^2^ = 0%). For macroscopic hematuria before biopsy (2 studies, *n* = 1264), the pooled RR was 0.60 (95% CI, 0.41–0.87; *p* = .008; I^2^ = 0%) (Table 2).

### Persistent hematuria and ESRD

Three studies [8,34,35] assessed the association between persistent hematuria (TA-hematuria) and ESRD or a 50% decline in eGFR. However, due to the different definitions and calculation methods of TA-hematuria, the pooled analysis was not applied. The latest retrospective cohort of 1333 IgAN patients [33] showed that the TA-hematuria (per 1 unit increase after logarithmic transformation) during follow-up was an independent predictor for ESRD or a 50% decline in eGFR (HR, 1.46; 95% CI, 1.13–1.87; *p* = .003). Similar result was observed in another chinese cohort [8]. The corresponding HR was 2.1 (95% CI, 1.6–2.7; *p* < .001) in multivarite Cox model. Additionally, the study of Sevillano et al. [35] demonstrated that TA-hematuria (per 1 unit increase) was associated with a higher risk of ESRD (HR, 2.84; 95% CI, 1.06–7.30; *p* = .04).

### Initial hematuria and renal progression

Three studies were identified to explore the association between hematuria and renal progression. A Chinese retrospective cohort [36] with 82 asymptomatic IgAN patients demonstrated that hematuria at renal biopsy was an independent risk factor for renal prognosis, which was defined as doubling of the SCr level (OR, 2.97; 95% CI, 1.34–5.13). A Japanese study [37] with 790 IgAN patients aimed to evaluate the magnitude of mild hematuria at biopsy for deterioration of renal function (doubling of SCr level). Multivariable logistic regression showed that the presence of mild hematuria was a significant predictor (OR, 2.3; 95% CI, 1.2–4.3). A Kuwait study [38] with 69 IgAN patients suggested that deterioration of renal function during the follow-up period (mean follow-up duration, 3.5 years) was more significant in the presence of macroscopic hematuria at the time of biopsy (*p* < .05). The crude RR was 1.47 (95% CI, 0.90–2.40).

## Discussion

Primary findings of our systematic review were as follows: (a) initial hematuria was not associated with a high risk of ESRD (RR, 1.32; 95% CI, 0.87–2.00; *p* = .19); (b) initial microscopic hematuria was associated with an 87% increased risk (RR, 1.87; 95% CI, 1.40–2.50; *p* < .001), while initial macroscopic hematuria was associated with a 32% decreased risk (RR, 0.68; 95% CI, 0.58–0.79; *p* < .001) for ESRD; (c) persistent hematuria might be an independent risk factor for poor renal outcomes (ESRD or 50% decline in eGFR) of IgAN patients.

Microscopic hematuria is defined by the presence of more than 3 RBCs per high-power field in urine sediment in the absence of colored urine. Macroscopic hematuria is always pathologic and is characterized by the massive presence of RBCs in urine. Isolated hematuria at the time of biopsy possibly enhances the sensitivity for the early detection of IgAN and might defines a cohort with a higher risk of disease progression appropriate for recruitment into clinical therapeutic trials within realistic time frames. Although glomerular hematuria has been considered a clinical manifestation of glomerular diseases without real consequences on renal function and long-term prognosis, up to 25% of patients with macroscopic hematuria-associated AKI do not recover baseline renal function for obstruction by red blood cell casts [39]. Therefore, the association between isolated microscopic hematuria in IgAN and the long-term incidence of end-stage renal disease needs to be explained and described clearly [40]. However, information about the long-term outcome of IgAN patients presenting with minor or benign clinical presentations is scarce. One reason is that few nephrological departments maintain the policy of renal biopsy performance in patients with minor urinary abnormalities, including persistent microscopic hematuria with or without minimal proteinuria, although a significant proportion of patients in whom a renal biopsy later establishes the diagnosis of IgAN can present with these minor manifestations. According to the data of these centers, less than half of patients with normal renal function, microalbuminuria, and without hypertension at the time of renal biopsy will develop more proteinuria, 26%–38% will develop hypertension, and 7%–24% will develop impaired renal function after a median follow-up of 7–11 years. These results suggest that IgAN is a progressive disease in a relevant fraction of patients, even in those with a more benign clinical presentation [5]. In conclusion, remission of hematuria may have a significant favorable effect on IgA nephropathy outcomes.

In our systematic review and meta-analysis, although IgAN patients with initial hematuria did not show significantly worse renal outcomes than those without, other hallmarks related to hematuria classification are still worthy of attention. Initial microscopic hematuria seems to be an important predictor for worsening renal outcomes. More interestingly, patients with persistent hematuria may more easily reach ESRD or 50% decline in renal function than those with minimal or negative hematuria. But a topic worthy of further study is how to use such a parameter, that dynamically reflects the disease status of IgAN patients over the whole course, to achieve early prediction of long-term prognosis.

On the other hand, some studies have further revealed that remission of hematuria may delay the progression of renal function and reduce the occurrence of adverse renal outcomes [34,35]. Unfortunately, spontaneous remission of hematuria and proteinuria are currently uncommon, and worse, there is insufficient data to support that remission of hematuria with a specific (and yet unknown) treatment could lead to a better long-term renal outcome [5,41]. Additionally, we are suprised to observe that IgAN patients with gross hematuria possess a decreased risk and better renal outcomes compared with those without any forms of hematuria or with microscopic hematuria. However, the deeper reasons and mechanisms are still pending. This requires more clinical evidence to confirm this phenomenon and explain its guiding significance for clinical practice.

To the best of our knowledge, this was the first meta-analysis that focused on the associations between hematuria and renal outcomes among IgAN patients. Literature screening, data extraction, and quality assessment were conducted in duplicate by 2 independent investigators. The analysis procedure was based on a rigorous, standardized, and previously-defined meta-analytic methodology. More importantly, in most groups, the between-study heterogeneity was not statistically significant, which increased the precision and power of the pooled estimate.

However, there are still several limitations in our work. First, most of the eligible studies were retrospective. The introduction of unblinded explorations might lead to an overestimation of the real result. Second, the nature of acquiring summary estimates and inaccessibility of individual patient data limit methods of controlling for confounding in the course of the meta-analysis. Nevertheless, the overwhelming majority of included publications controlled adequately for confounding during modeling of outcomes, reducing the risk for residual confounding. Third, we noted that the heterogeneity in the initial hematuria group was moderate. In subgroup analyses, we noticed that the classification of hematuria, protenuria quantification, and initial eGFR value could explain a part of the heterogeneity. Unfortunately, owing to the limited number of studies, we were unable to further explore the other sources of heterogeneity. Similarly, the number of qualified studies restricted the use of sensitivity analyses and publication bias tests. Additionally, a few articles could be missed as a result of the language limitation of our literature retrieval.

## Conclusion

In conclusion, initial microscopic hematuria or persistent hematuria was associated with a higher risk of ESRD, while initial macroscopic hematuria was a protective factor for ESRD among patients with IgAN, independent of other traditional predictors. Risk stratification of ESRD could consider various hematuria as significant predictors for long-term renal survival. Ultimately, randomized studies are needed to determine whether hematuria treatments in patients with decreased kidney function can improve the excess ESRD burden associated with the coexistence of these conditions.

## Appendices

### Appendix 1 Checklist of items in the meta-analysis according to PRISMA statement

**Table:**

Section/topic	Item No.	Checklist item	Page No.
**Title**			
Title	1	Identify the report as a systematic review, meta-analysis, or both	1
**Abstract**			
Structured summary	2	Provide a structured summary including, as applicable, background, objectives, data sources, study eligibility criteria, participants, interventions, study appraisal and synthesis methods, results, limitations, conclusions and implications of key findings, systematic review registration number	2
**Introduction**			
Rationale	3	Describe the rationale for the review in the context of what is already known	3
Objectives	4	Provide an explicit statement of questions being addressed with reference to participants, interventions, comparisons, outcomes, and study design	3
**Methods**			
Protocol and registration	5	Indicate if a review protocol exists, if and where it can be accessed, and, if available, provide registration information including registration number	4
Eligibility criteria	6	Specify study characteristics (such as PICOS, length of follow-up) and report characteristics (such as years considered, language, publication status) used as criteria for eligibility, giving rationale	4
Information sources	7	Describe all information sources (such as databases with dates of coverage, contact with study authors to identify additional studies) in the search and date last searched	4
Search	8	Present full electronic search strategy for at least one database, including any limits used, such that it could be repeated	4
Study selection	9	State the process for selecting studies (that is, screening, eligibility, included in systematic review, and, if applicable, included in the meta-analysis)	5
Data collection process	10	Describe method of data extraction from reports (such as piloted forms, independently, in duplicate) and any processes for obtaining and confirming data from investigators	5,6
Data items	11	List and define all variables for which data were sought (such as PICOS, funding sources) and any assumptions and simplifications made	5,6
Risk of bias in individual studies	12	Describe methods used for assessing risk of bias of individual studies (including specification of whether this was done at the study or outcome level), and how this information is to be used in any data synthesis	6
Summary measures	13	State the principal summary measures (such as risk ratio, difference in means)	6
Synthesis of results	14	Describe the methods of handling data and combining results of studies, if done, including measures of consistency (such as I^2^) for each meta-analysis	6,7
Risk of bias across studies	15	Specify any assessment of risk of bias that may affect the cumulative evidence (such as publication bias, selective reporting within studies)	6,7
Additional analyses	16	Describe methods of additional analyses (such as sensitivity or subgroup analyses, meta-regression), if done, indicating which were pre-specified	7
**Results**			
Study selection	17	Give numbers of studies screened, assessed for eligibility, and included in the review, with reasons for exclusions at each stage, ideally with a flow diagram	7
Study characteristics	18	For each study, present characteristics for which data were extracted (such as study size, PICOS, follow-up period) and provide the citations	7
Risk of bias within studies	19	Present data on risk of bias of each study and, if available, any outcome-level assessment	8
Results of individual studies	20	For all outcomes considered (benefits or harms), present for each study (a) simple summary data for each intervention group and (b) effect estimates and confidence intervals, ideally with a forest plot	7
Synthesis of results	21	Present results of each meta-analysis done, including confidence intervals and measures of consistency	8-10
Risk of bias across studies	22	Present results of any assessment of risk of bias across studies	8-10
Additional analysis	23	Give results of additional analyses, if done (such as sensitivity or subgroup analyses, meta-regression)	8-10
**Discussion**			
Summary of evidence	24	Summarise the main findings including the strength of evidence for each main outcome; consider their relevance to key groups (such as health care providers, users, and policy makers)	10
Limitations	25	Discuss limitations at study and outcome level (such as risk of bias), and at review level (such as incomplete retrieval of identified research, reporting bias)	13
Conclusions	26	Provide a general interpretation of the results in the context of other evidence, and implications for future research	13
**Funding**			
Funding	27	Describe sources of funding for the systematic review and other support (such as supply of data) and role of funders for the systematic review	14

### Appendix 2 Literature retrieval strategies

2020/5/14

**Pubmed**

((((((((IgA Nephropathy 1) OR Berger Disease) OR IgA Type Nephritis) OR Immunoglobulin A Nephropathy) OR IgA Nephropathy) OR IgA Glomerulonephritis) OR "Glomerulonephritis, IGA"[Mesh])) AND ((((hematuria) OR haematuria) OR "Hematuria"[Mesh]) AND ( "1990/01/01"[PDat] : "2020/05/14"[PDat] ))                 **1113**

**Embase**

No. Query Results                    Results Date

#13. #12 AND (1990:py OR 1991:py OR 1992:py OR 1993:py    **2,555** 14 May 2020 OR 1994:py OR 1995:py OR 1996:py OR 1997:py OR

  1998:py OR 1999:py OR 2000:py OR 2001:py OR

  2002:py OR 2003:py OR 2004:py OR 2005:py OR

  2006:py OR 2007:py OR 2008:py OR 2009:py OR

  2010:py OR 2011:py OR 2012:py OR 2013:py OR

  2014:py OR 2015:py OR 2016:py OR 2017:py OR

  2018:py OR 2019:py OR 2020:py) AND 'human'/de AND

  ('hematuria'/dm OR 'immunoglobulin a

  nephropathy'/dm)

**Table:**

#12. #7 AND #11	3,241	14 May 2020
#11. #8 OR #9 OR #10	57,269	14 May 2020
#10. Haematuria	8,697	14 May 2020
#9. hematuria	55,747	14 May 2020
#8. 'hematuria'/exp	49,550	14 May 2020
#7. #1 OR #2 OR #3 OR #4 OR #5 OR #6	32,055	14 May 2020
#6. berger AND disease	13,767	14 May 2020
#5. iga AND type AND nephritis	425	14 May 2020
#4. immunoglobulin AND a AND nephropathy	16,671	14 May 2020
#3. iga AND nephropathy	10,193	14 May 2020
#2. iga AND glomerulonephritis	5,731	14 May 2020
#1. 'immunoglobulin a nephropathy'/exp	11,360	14 May 2020

### Appendix 3 Quality assessment of the included studies

**Table:**

References	Representa- tiveness^a^	Selection of non-exposed^b^	Ascertainment of exposure^c^	Incident disease^d^	Comparabi-lity^e^	Outcome assessment^f^	Length of follow-up^g^	Adequacy of follow up^h^	Quality score
De Menezes 2020 [22]	B	A	A	B	B	B	A	D	6
Bobart 2019 [23]	A	A	A	B	B	B	B	C	5
Heybeli 2019 [24]	A	A	A	B	B	B	A	C	6
Tanaka 2015 [14]	B	A	A	B	A + B	B	B	D	6
Le 2014 [25]	A	A	A	B	B	B	A	D	6
Bjørneklett 2012 [27]	A	A	A	B	A + B	B	A	D	7
Lee 2012 [26]	A	A	A	B	B	B	A	B	7
Goto 2009 [9]	A	A	A	A	A + B	B	A	C	8
Espinosa 2009 [28]	A	A	A	B	B	B	B	D	5
Manno 2007 [29]	A	A	A	B	A + B	B	A	C	7
Daniel 2000 [30]	A	A	A	B	A + B	C	B	D	5
Haas 1997 [31]	A	A	A	B	B	B	B	C	5
Frimat 1997 [32]	A	A	A	A	B	A	A	C	7

^a^A, truly representative; B, somewhat representative; C, selected group; D, no description of the derivation of the cohort.

^b^A, drawn from the same community as the exposed; B, drawn from a diferent source; C, no description of the derivation of the non-exposed.

^c^A, secure record; B, structured interview; C, written self-report; D no description.

^d^Demonstration that the outcome of interest was not present at start of study: A yes, B no.

^e^A, study controls for demographics/comorbidities; B, study controls for any additional factor; C not done.

^f^A, independent or blind assessment; B, record linkage; C, self-report; D, no description.

^g^Long enough for outcomes to occur? A, yes (≥ 5 yrs); B, no (< 5 yrs).

^h^A, complete follow-up; B, subjects lost to follow-up was unlikely to introduce bias; C, follow-up rate 90% or lower; D no statement.

### Appendix 4 Sensitivity analyses through the jackknife approach

**Table:**

Authors/Publication years	Study excluded, pooled RRs	Study excluded, 95% CI
**Initial hematuria**		
De Menezes (2020)	1.42	0.90 to 2.25
Bobart (2019)	1.31	0.85 to 2.03
Bjørneklett (2012)	1.27	0.77 to 2.11
Goto (2009)	1.16	0.78 to 1.74
Manno (2007)	1.20	0.74 to 1.95
Daniel (2000)	1.54	1.06 to 2.22
Frimat (1997)	1.35	0.85 to 2.15
**Initial macroscopic hematuria**		
Heybeli/2019	0.60	0.49 to 0.74
Tanaka/2015	0.69	0.60 to 0.80
Le/2014	0.70	0.60 to 0.81
Lee/2012	0.72	0.63 to 0.82
Goto/2009	0.73	0.64 to 0.82
Espinosa/2009	0.72	0.63 to 0.82
Haas/1997	0.70	0.61 to 0.80

CI, confidence interval; RR, relative risk.
